# Effects of uric acid on ischemic diseases, stratified by lipid levels: a drug-target, nonlinear Mendelian randomization study

**DOI:** 10.1038/s41598-024-51724-1

**Published:** 2024-01-16

**Authors:** Jungeun Kim, Sun Yeop Lee, Jihye Lee, Sanghyuk Yoon, Eun Gyo Kim, Eunbyeol Lee, Nayoung Kim, Sol Lee, Ho Gym, Sang-In Park

**Affiliations:** 1 Basgenbio Inc., Seoul, Republic of Korea; 2 Department of Statistics and Actuarial Science, College of Natural Sciences, Soongsil University, Seoul, Republic of Korea; 3 Department of Epidemiology and Health Promotion, Graduate School of Public Health, Yonsei University, Seoul, Republic of Korea; 4 Department of Pharmacology, College of Medicine, Kangwon National University, 1 Gangwondaehak-gil, Chuncheon-si, Gangwon-do 24341 Republic of Korea; 5 Biomedical Research Institute, Kangwon National University Hospital, Chuncheon, Republic of Korea

**Keywords:** Computational biology and bioinformatics, Genetics, Cardiology, Medical research

## Abstract

Although uric acid-lowering agents such as xanthine oxidase inhibitors have potential cardioprotective effects, studies on their use in preventing cardiovascular diseases are lacking. We investigated the genetically proxied effects of reducing uric acid on ischemic cardiovascular diseases in a lipid-level-stratified population. We performed drug-target Mendelian randomization (MR) analyses using UK Biobank data to select genetic instruments within a uric acid-lowering gene, xanthine dehydrogenase (*XDH*), and construct genetic scores. For nonlinear MR analyses, individuals were stratified by lipid level. Outcomes included acute myocardial infarction (AMI), ischemic heart disease, cerebral infarction, transient cerebral ischemic attack, overall ischemic disease, and gout. We included 474,983 non-gout individuals with *XDH*-associated single-nucleotide polymorphisms. The *XDH*-variant-induced uric acid reduction was associated with reduced risk of gout (odds ratio [OR], 0.85; 95% confidence interval [CI], 0.78–0.93;* P* < 0.001), cerebral infarction (OR, 0.86; 95% CI, 0.75–0.98; *P* = 0.023), AMI (OR, 0.79; 95% CI, 0.66–0.94; *P* = 0.010) in individuals with triglycerides ≥ 188.00 mg/dL, and cerebral infarction in individuals with low-density lipoprotein cholesterol (LDL-C) ≤ 112.30 mg/dL (OR, 0.76; 95% CI, 0.61–0.96; *P* = 0.020) or LDL-C of 136.90–157.40 mg/dL (OR, 0.67; 95% CI, 0.49–0.92; *P* = 0.012). *XDH*-variant-induced uric acid reduction lowers the risk of gout, AMI for individuals with high triglycerides, and cerebral infarction except for individuals with high LDL-C, highlighting the potential heterogeneity in the protective effects of xanthine oxidase inhibitors for treating AMI and cerebral infarction depending on the lipid profiles.

## Introduction

Cardiovascular diseases (CVDs) are a leading cause of premature mortality and disability worldwide^1^. The leading causes of death are ischemic heart disease (IHD) and stroke, accounting for 16% and 11% of global deaths, respectively^2^. Modifiable risk factors for CVD include hypertension, smoking, obesity, and aberrant cholesterol levels, which tend to cluster and can synergistically increase CVD risk^3^.

Increased serum uric acid is associated with increased CVD risk^4^. However, there are conflicting results regarding their causal relationship. For example, the Framingham Heart Study demonstrated that uric acid level was not associated with increased risk for cardiovascular events after adjusting for risk factors or confounders^5^. Evidence also suggests that confounding factors, such as body mass index (BMI), also account for the associations between uric acid level and CVD risk^6^.

Xanthine oxidase inhibitors (XOIs) inhibit xanthine oxidoreductase, which has two interconvertible forms, xanthine oxidase and xanthine dehydrogenase (XDH)^7^. In addition to its therapeutic effects on gout, observational studies of allopurinol, a purine-like XOI, have suggested its cardioprotective effects, including reduced myocardial infarction and stroke risks^8,9^. In contrast, the ALL-HEART study, a recent multicenter randomized controlled trial (RCT) in patients with IHD without a history of gout, found no benefits of allopurinol therapy (compared to usual care) for the primary outcomes of non-fatal myocardial infarction, non-fatal stroke, or cardiovascular-related death^10^. Despite the RCT findings, the cardiovascular benefits of XOIs in different contexts remain unclear^11^, especially considering the robust mechanistic understanding of xanthine oxidoreductase inhibition in vascular damage^12^. Moreover, the ALL-HEART study was limited to participants with IHD (primarily long-term), with no history of gout, with the control group receiving the usual care for IHD. Therefore, extrapolating its findings to populations with acute IHD or those with different levels of gout or CVD-related risk factors, such as symptomatic hyperuricemia or elevated cholesterols, may not be possible. RCTs and observational studies have reported meaningful signals for clinical outcomes in the subgroup stratified by uric acid levels or CVD risk factors^13,14^. However, RCTs have typically compared different XOIs in patients with gout without a placebo group^13,15^ or were limited to individuals without gout^16,17^.

Ischemic stroke and IHD share risk factors and have a similar mechanism: atherosclerosis^18^. Dyslipidemia is a major modifiable risk factor for ischemic stroke and IHD. Due to its association with pathogenesis in atheroma, hyperuricemia is related to dyslipidemia, suggesting its role in atherosclerosis development^19^. Nonetheless, no prior studies have quantitatively evaluated the relationship between serum uric acid and CVD across different lipid profiles. Nonlinear Mendelian randomization (MR) can be used to explore the potentially heterogeneous effects of XOIs across subgroups^20^. However, MR studies using uric acid-associated genetic instrumental variables (IVs) to assess the causal relationships between serum uric acid and CVD have yielded mixed findings^21,22^. These studies have used genetic IVs from multiple gene regions (a polygenic approach) rather than focusing on one gene (by drug-target MR) and assessed only average effects in a linear MR framework^21,22^.

There is an important difference between using genetically proxied uric acid from the polygenic effects versus using *XDH*-variant-proxied uric acid from the cis-variant effect. To address these gaps in knowledge, we performed a linear drug-target MR analysis to investigate the effects of reducing uric acid levels by XDH inhibition on the risk of ischemic diseases in individuals whose data are in the UK Biobank. Furthermore, we stratified populations by uric acid and lipid levels to explore potentially heterogeneous effects.

## Results

### Study population

Following quality control, 486,624 participants in the UK Biobank were included (Supplementary Table S1) [mean (SD) age, 56.54 (8.09); uric acid, 5.20 (1.35) mg/dL, LDL-C, 137.53 (33.64) mg/dL; TG, 154.78 (91.00) mg/dL; HDL-C, 55.99 (14.78) mg/dL; and TC/HDL-C ratio, 4.24 (1.23)]. In total, 23,094 gout cases and 88,843 overall ischemic disease cases were included. For the genome-wide association study (GWAS), 11,641 patients with gout before uric acid measurement were excluded from selecting genetic IVs for *XDH*. For nonlinear MR, the mean values per quartile were 3.89, 4.70, 5.44, and 6.76 mg/dL for uric acid; 96.63, 125.29, 146.98, and 181.21 mg/dL for LDL-C; 78.97, 110.02, 154.99, and 275.15 mg/dL for TG; and 2.99, 3.68, 4.42, and 5.86 for the TC/HDL-C ratio (Supplementary Table S2).

### Genetic IV selection

Within ± 500 kb of *XDH* after clumping, six single-nucleotide polymorphisms (SNPs) were associated with serum uric acid at the gene-specific Bonferroni significance level, and three with serum uric acid at the genome-wide significance level (Supplementary Table S3), regardless of the clumping threshold (*r*^*2*^ < 0.1 or 0.3). Potentially pleiotropic SNPs were excluded, resulting in three SNPs at the gene-specific significance threshold and one (also the top SNP) at the genome-wide significance threshold. The excluded SNPs were associated with *NLRC4*, *IL-18*, *SLC30A6*, *YIPF4*, and *CAPN13* expression, possibly associated with CVD-related factors. Rather than using one SNP for *XDH* genetic scoring, three were used based on their *F*-statistic (22.81), twice the explained variance (0.02%), and stability in constructing the genetic score. The final three SNPs or the genetic score showed null associations with age and sex, suggesting that the collider bias may have had a negligible effect on the IVs (Supplementary Table S4).

### Linear MR: causal effects of uric acid on gout and ischemic diseases

**Figure 1:**
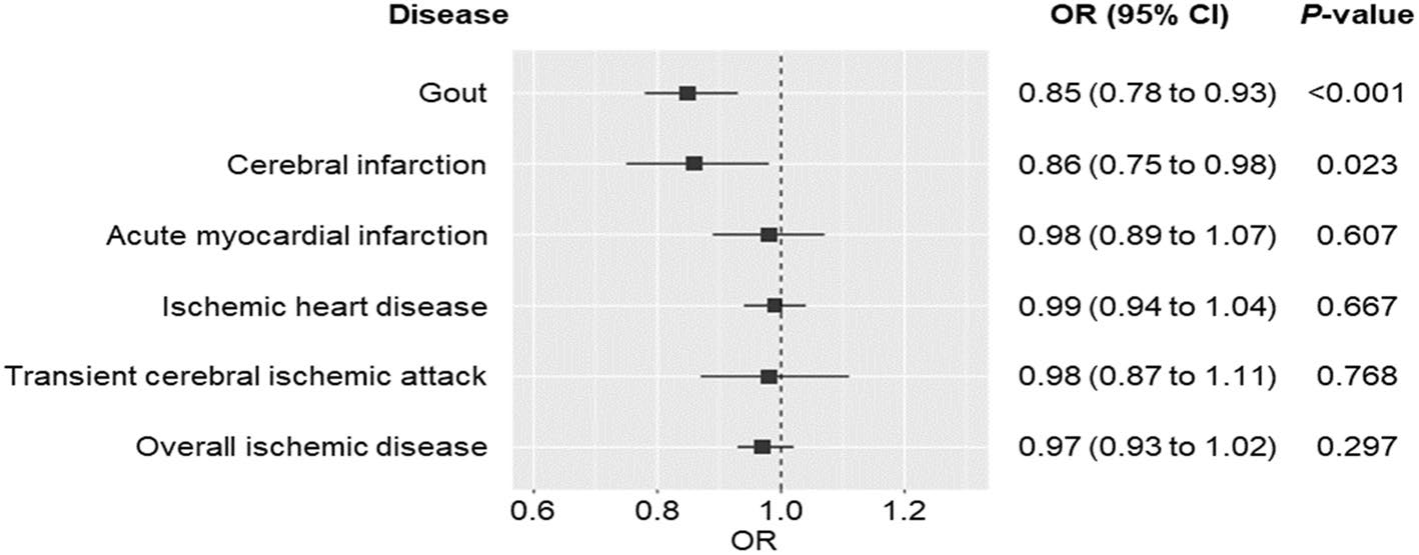
Linear Mendelian randomization results for gout and ischemic diseases. OR: odds ratio, CI: confidence interval.

A one-unit decrease in the scaled serum uric acid levels related to the selected *XDH* variants was associated with a 15% reduction in the odds of gout (odds ratio [OR], 0.85; 95% confidence interval [CI], 0.78–0.93; *P* < 0.001) and cerebral infarction (OR, 0.86; 95% CI, 0.75–0.98; *P* = 0.023). In contrast, the *XDH*-variant-induced reduction in serum uric acid was not significantly associated with lower odds of AMI (OR, 0.98; 95% CI, 0.89–1.07; *P* = 0.607), IHD (OR, 0.99; 95% CI, 0.94–1.04; *P* = 0.667), transient cerebral ischemic attack (OR, 0.98; 95% CI, 0.87–1.11; *P* = 0.768), or overall ischemic disease (OR, 0.97; 95% CI, 0.93–1.02; *P* = 0.297) (Fig. 1). The association between the *XDH* genetic score and BMI, a potential confounder, was null (β, SE: − 0.16, 0.45; *P* = 0.726), and findings of linear MR for all diseases remained unchanged after additionally adjusting for BMI (Supplementary Table S5). Furthermore, the findings were consistent with those obtained using the MR based on a constrained maximum likelihood (MR-cML) method (Supplementary Table S5).

### Nonlinear MR: subgroup analyses by uric acid and lipid levels

The *XDH* genetic score had a statistically significant effect on uric acid (*β*, SE: − 1.01, 0.01; *P* < 0.001) (Supplementary Table S2) but not on LDL-C (*β*, SE: − 0.14, 3.21; *P* = 0.964), TG (*β*, SE: 14.26, 8.50; *P* = 0.093), HDL-C (*β*, SE: 1.70, 1.34; *P* = 0.206), or TC/HDL-C (*β* < 0.01; SE, 0.12; *P* = 0.991), providing additional evidence against pleiotropy and collider bias in our subgroup analyses. The doubly ranked or residual-based methods detected no violations of the constant genetic effect assumption.

**Figure 2:**
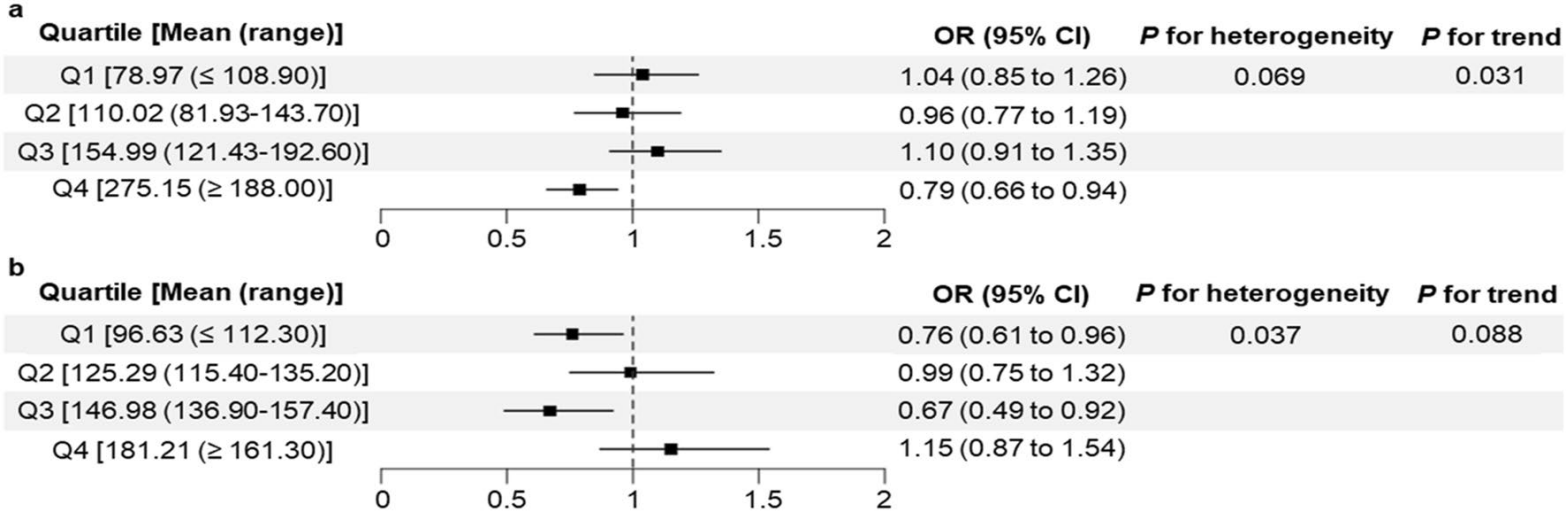
Nonlinear Mendelian randomization results for (**a**) acute myocardial infarction stratified by triglyceride levels and for (**b**) cerebral infarction stratified by low-density lipoprotein cholesterol levels. The ranges of observed values may overlap across quartiles because the quartiles were stratified based on residuals, not the observed values. OR: odds ratio, CI: confidence interval.

The associated reductions in serum uric acid and gout risk were similar across the subgroups stratified by serum uric acid or lipid levels, with no trends or heterogeneity (Supplementary Table S6). For AMI, nonlinearity across TG-stratified subgroups was observed (*P*_*trend*_ = 0.031) (Fig. 2; Supplementary Table S7). The *XDH*-variant-induced reduction in serum uric acid was significantly associated with reduced odds of AMI (OR, 0.79; 95% CI, 0.66–0.94; *P* = 0.010) in individuals with TG ≥ 188.00 mg/dL (Q4). For cerebral infarction, heterogeneity across LDL-C-stratified subgroups was observed (*P*_*heterogeneity*_ = 0.037). The *XDH*-variant-induced reduction in serum uric acid was significantly associated with reduced odds of cerebral infarction in individuals with LDL-C ≤ 112.30 mg/dL (Q1) (OR, 0.76; 95% CI, 0.61–0.96; *P* = 0.020) and LDL of 136.90–157.40 mg/dL (Q3) (OR, 0.67; 95% CI, 0.49–0.92; *P* = 0.012). When stratified by HDL-C, the risks of IHD and overall ischemic disease were significantly reduced in the HDL-C ≤ 50.62 mg/dL subgroup (Q1); however, these risks were higher in the 49.23–67.25 mg/dL subgroup (Q3) (Supplementary Tables S6 and S8). No heterogeneity was observed for the effect of the *XDH*-variant-induced reduction in serum uric acid on the risk of transient cerebral ischemic attack.

## Discussion

To our knowledge, this is the first drug-target MR study to focus on the effect of XDH inhibition on uric acid. In this study, the *XDH*-variant-induced reduction in serum uric acid was associated with reduced cerebral infarction and gout risks. The effect of uric acid reduction on gout mediated by *XDH*-inhibition was maintained following stratification by lipid level. For CVD, the *XDH*-variant-induced reduction in serum uric acid was not significantly associated with ischemic disease outcomes, except for cerebral infarction. In contrast, following lipid-based stratification, the AMI risk was significantly reduced in the TG ≥ 188.00 mg/dL subgroup (Q4), and that of cerebral infarction was significantly reduced in the LDL ≤ 112.30 mg/dL (Q1) and LDL 136.90–157.40 mg/dL (Q3) subgroups.

Since their positive association has been well established^23^, we first confirmed the causal association between serum uric acid level and the risk of gout as a positive control outcome. Additionally, lower serum uric acid levels were causally associated with a decreased risk for cerebral infarction, consistent with previous findings^24,25^. Hyperuricemia associated with uric acid deposition is a risk factor for IHD and stroke^26,27^. Although the pathophysiological role of uric acid in CVD development remains unclear, several plausible mechanisms have been proposed, including elevated serum uric acid-associated inflammation, oxidative stress, insulin resistance, endothelial dysfunction, and platelet activation, which affect CVD and atherosclerosis progression^28^. However, neither a recent large RCT^10^ nor an umbrella review^23^ supports the therapeutic benefits of uric acid-reducing agents in CVD prevention. Therefore, we hypothesized that the protective effects of XOIs against CVD might be limited in certain populations, depending on their CVD risk factors.

Hyperuricemia is common in individuals with dyslipidemia^26^, and a significant association was observed among dyslipidemia, hyperuricemia, and obesity^29^. Possible mechanisms have been suggested to explain the relationships between hyperuricemia and dyslipidemia. For example, elevated levels of serum uric acid significantly predicted the presence of smaller, denser LDL-C and HDL-C particles, which are associated with a higher atherogenic risk^30^. In addition, hyperuricemia can affect adipocytes by increasing monocyte chemotactic protein levels and reducing the production of adiponectin, thereby contributing to insulin resistance and inflammation^31,32^. Previous studies have reported a linear relationship between serum uric acid and serum LDL-C and TG, with an inverse relationship between serum uric acid and HDL-C^32,33^. Based on these findings, various types of lipids may affect the association between serum uric acid and CVD outcomes in different ways.

A nonlinear relationship between serum uric acid and CVD risk has been reported, with a J-shaped trend between serum uric acid and stroke risk^34^. Furthermore, a dose-dependent association between serum uric acid and coronary heart disease incidence was observed, but only for individuals without comorbidities such as hypertension and metabolic syndromes^35^. Therefore, analyzing nonlinear relationships between serum uric acid and ischemic disease outcomes in the context of MR may elucidate their associations, particularly for individuals with dyslipidemia. To accomplish this, we stratified populations by lipid levels to explore potentially heterogeneous effects.

While there were no significant associations between the uric acid-reducing effect of XDH inhibition and AMI risk, this risk was significantly reduced in the highest serum-TG quantile (Q4). Similarly, for IHD and overall ischemic disease, for which no average effect was found, a significant association was observed for the lowest HDL-C quantile; however, the heterogeneity among subgroups disappeared when stratified by the TC/HDL-C ratio. Meanwhile, a reduced risk for cerebral infarction was observed in the lower LDL-C subgroups, but this risk was not significantly reduced in the highest LDL-C quantile (Q4). Considering the harmful impact of high LDL-C in ischemic stroke (particularly atherothrombotic stroke)^36^, the protective effect of reducing uric acid may not occur in the highest-LDL-C subgroup. These findings suggest that the potential benefits of XOI inhibitors for reducing the risks of ischemic disease may vary depending on the lipid profile.

Our study has some limitations. First, the residual-biomarker approach to nonlinear MR analysis, and the number of lipid subtypes used for stratification, led to multiple testing and an increased risk of type 1 errors. Nonetheless, the somewhat consistent results across the lipid subtypes provide additional support. Second, MR analysis cannot provide differential results for drugs with the same target, such as allopurinol and febuxostat. Third, since few of the *XDH* variants in the UK Biobank were genotyped in the commonly used extensive, external GWAS summary data for uric acid (i.e., Global Urate Genetics Consortium), we were unable to use externally derived weights for the genetic score. Nevertheless, using individual-level data enabled us to ensure (1) the temporality of the relationship between the exposure (i.e., uric acid) and the outcome (i.e., gout and CVDs), and (2) consistency in covariate adjustments in the genetic associations with exposures or outcomes, which are often ignored in MR studies that use externally derived weights. Fourth, MR estimates must be interpreted as the lifelong effect of genetically determined uric acid level and cannot be directly compared to RCT estimates. RCT estimates are different from MR estimates in that they represent short-term effects of intervention and are dependent on dosage. MR estimates are also subject to canalization, the process in which genetically determined levels of a trait are compensated by developmental processes^37^. Future RCTs are required to understand the true nonlinear relationships between uric acid and ischemic diseases. Fifth, nonlinear MR is a relatively new method with limitations. Specifically, methodological concerns have been raised recently; the constant genetic effect assumption is sometimes unreasonable, and stratification by exposure may lead to residual selection bias^38^. However, we carefully examined these issues with the application of the doubly ranked method and the negative outcome analyses, and concluded that these issues did not impact our findings.

Despite these limitations, our study presents comprehensive, genetically predicted results for XOIs, providing a novel strategy for reducing ischemic disease risk, particularly in patients with higher (or lower) lipid burdens. However, validating these findings will require further prospective RCTs to evaluate the effects of uric acid-reducing agents combined with lipid-reducing agents, considering various factors affecting CVD risk.

In conclusion, this drug-target MR study demonstrated that *XDH*-variant-induced reductions in serum uric acid were significantly associated with reduced risks of cerebral infarction and gout. Following stratification by lipid levels, XDH inhibition exhibited significant protective effects against AMI and IHD in the highest-risk TG and HDL-C subgroups, respectively, with no effect on cerebral infarction in the highest LDL-C subgroup. Our findings suggest that XOIs may interact with patients’ lipid profiles to produce heterogeneous effects and that there may be potential benefits of using XOIs for ischemic disease prevention in subpopulations with dyslipidemia.

## Methods

### Study population and ethical approval

We retrieved data from the UK Biobank (https://www.ukbiobank.ac.uk/enable-your-research/apply-for-access; Application Number 77890) to select genetic IVs in *XDH*, a target of XOI, and determine the effect of XDH inhibition on disease outcomes. The UK Biobank obtained approval from the Northwest Multicenter Research Ethics Committee (REC reference: 21/NW/0157). All methods were carried out in accordance with relevant guidelines and regulations^39^. All participants provided written informed consent. Participants were aged 37–73 years at data collection. There was no experimental protocol requiring prior institutional and/or licensing committee approval since we used anonymized, open-source data.

### Genetic IV selection

Serum uric acid was considered as an exposure when mimicking the effect of XOIs. A GWAS was performed to identify SNPs related to serum uric acid. First, uric-acid-associated SNPs within a ± 500 kb range of the XDH gene were selected at three candidate significance levels: the top SNP (i.e., the SNP with the lowest *P*-value), the genome-wide significance level (*P* < 5.00 × 10^−8^), and the gene-specific Bonferroni-adjusted significance level (*P* < 9.47 × 10^−6^; 0.05/5279). SNPs with linkage disequilibrium were clumped at two different thresholds (*r*^*2*^ < 0.1 or 0.3) using the 1000 Genomes European reference panel^40^ to include only independent SNPs. Next, potentially pleiotropic SNPs were excluded if they or their proxies were associated with outcome-related traits (i.e., gene expression, proteins, metabolites, biomarkers, or diseases) at linkage disequilibrium (*r*^*2*^ > 0.8; ± 500 kb) based on public databases (GTExPortal, https://www.gtexportal.org/home/; the GWAS catalog, https://www.ebi.ac.uk/gwas/; and PhenoScanner, http://www.phenoscanner.medschl.cam.ac.uk/). After removing potentially pleiotropic SNPs, their IV strengths (*F*-statistic) and amounts of variance explained (*R*^*2*^) were compared for all candidate scenarios (i.e., the three *P*-value thresholds and two *r*^*2*^-based clumping thresholds) to select the final IV set. Finally, a weighted genetic score was calculated using the SNPs in the final IV set and was used as an IV for all following analyses. For sensitivity analysis, the associations between the genetic IVs and negative control outcomes, age, and biological sex, were assessed. Non-null associations may indicate the presence of collider bias arising from self-selection into the dataset.

### Outcomes

The initial efficacy outcome for XOI was gout, a positive control outcome under the drug-target MR framework. This association was confirmed to improve the credibility of the selected genetic IVs for use in the drug-target MR analysis to investigate the potential for XOI repurposing.

The CVD outcomes of interest in this study were several ischemic diseases (acute myocardial infarction [AMI], IHD, cerebral infarction, and transient cerebral ischemic attack) and a composite of these outcomes (i.e., overall ischemic disease). Samples with these outcomes were defined according to the disease categories of the International Classification of Diseases, 10th revision (ICD-10) (Supplementary Table S9). Control status was defined using Phecode version 1.2, which minimizes the case-contamination noise prevalent in ICD − 10 codes^41^.

### Statistical analyses

For GWAS, linear regression analysis was performed using uric acid level as a dependent variable, with age, sex, five principal components of ancestry, genotyping array, and assessment center as covariates. Individuals diagnosed before uric acid measurement were excluded to prevent the prescribed medication from interfering with the SNP-uric acid association prediction. For additional quality control, those without genotyping data, those with a missing or implausible date of gout diagnosis (e.g., before birth), those with mismatched sex between genetic and self-reported sex, and those with genetic kinship to other participants (i.e., ten or more third-degree relatives identified) were excluded.

For linear MR, we estimated the causal effect of XDH-inhibition-induced reduction in serum uric acid using the Wald ratio estimation (i.e., the ratio-of-coefficients method) using a weighted genetic score as a single IV. Coefficients (*β*) obtained from the uric acid GWAS were used to calculate weighted genetic scores for all individuals, as follows:

$${Genetic\, Score}_{i}={\beta }_{1}{g}_{i1}+\dots +{\beta }_{k}{g}_{ik}$$

where *i* refers to the individual (*i* = 1, …, *n*), *k* refers to each genetic variant (*g*_*i*1_, …, *g*_*ik*_), *β* indicates the effect size of genetic variant *k*, and *g* indicates the number of exposure alleles (i.e., *g* = 0, 1, 2) for genetic variant *k* of individual *i*. The analysis scale was adjusted to 0.1 mg/dL of uric acid. The Wald ratio for each outcome was calculated by dividing the effect of the genetic score on each outcome by the effect of the genetic score on serum uric acid. Using the weighted genetic score for the Wald ratio is asymptotically equivalent to using multiple uncorrelated IVs for the inverse-variance-weighted method, the most statistically efficient method under the assumption that all IVs used are valid^42^. The covariates in the GWAS for uric acid were included in these effect estimations. For each outcome, those with a missing or implausible date of outcome diagnosis (e.g., before birth) were excluded, as in the GWAS for uric acid.

Sensitivity analyses for horizontal pleiotropy were conducted in two ways–a biologically driven approach and a statistically driven approach. First, for the biologically driven approach, the association between the genetic score and BMI was estimated, and linear MR was re-run by adjusting for BMI. BMI is a potential confounder in the relationship between uric acid and ischemic diseases^6^. Second, for the statistically driven approach, the linear MR was repeated using MR-cML. MR-cML has been recently developed to adjust for potential correlated and uncorrelated pleiotropy and shown to outperform other methods in a range of simulations^43^.

For nonlinear MR, individuals were stratified based on the quartiles of the lipid biomarkers (LDL-C, high-density lipoprotein cholesterol [HDL-C], total cholesterol/HDL-C [TC/HDL-C ratio], and triglyceride [TG]). Stratification based on observed biomarker levels may induce collider bias if the genetic score affects these biomarkers^20^. Biomarker residuals were calculated by subtracting the genetic score effect and effects of the covariates included in the GWAS on each biomarker from its observed value. Quartile-based stratification was chosen to ensure that a sufficient number of cases remained in each stratum. Before estimating the causal effect, the assumption of constant genetic effect embedded in the residual-based method (i.e., that the genetic effect on serum uric acid is linear and constant for all individuals) was tested using the doubly-ranked method, as recommended^38^. The effect of the genetic score on each biomarker was presented to check for potential pleiotropy through the lipid profiles. The Wald ratio was calculated within each stratum to estimate stratum-specific causal effects of XDH-inhibition-induced reduction in serum uric acid. The nonlinearity of the stratum-specific effects was tested using the heterogeneity test (Cochran’s Q statistic) and the quadratic-trend test.

PLINK version 1.9^44^ was used for GWAS. For MR analysis, the packages *TwoSamplMR*^45^, *MendelianRandomization*^46^, and *SUMnlmr*^47^ for R (version 4.0.5; The R Foundation, Vienna, Austria) were used. Details of the present study are reported according to a reporting guideline for an MR study design, namely, STROBE-MR^48^.

### Supplementary Information

Supplementary Information.

## Data Availability

The data used are available from the UK Biobank under the arrangements detailed at https://www.ukbiobank.ac.uk/enable-your-research/apply-for-access.
