# Supplementary material for: Effects of uric acid on ischemic diseases, stratified by lipid levels: a drug-target, nonlinear Mendelian randomization study
Source: Sci Rep. 2024 Jan 16;14:1338. doi: 10.1038/s41598-024-51724-1 (PMC10791707; doi:10.1038/s41598-024-51724-1)
Supplement: Supplementary file 1 — Supplementary Information. [file 41598_2024_51724_MOESM1_ESM.docx]

**Effects of uric acid on ischemic disease, stratified by lipid levels: a drug-target, nonlinear Mendelian randomization study**

**Jungeun Kim**^1, 2#^, **Sun Yeop Lee**^1#^, **Jihye Lee**^1^, **Sanghyuk Yoon**^1, 3^, **Eun Gyo Kim**^1^, **Eunbyeol Lee**^1^, **Nayoung Kim**^1, 3^, **Sol Lee**^1^, **Ho Gym**^1^, **and** **Sang-In Park**^4, 5*^

^1^Basgenbio Inc., Seoul, Republic of Korea

^2^Department of Statistics and Actuarial Science, College of Natural Sciences, Soongsil University, Seoul, Republic of Korea

^3^Department of Epidemiology and Health Promotion, Graduate School of Public Health, Yonsei University, Seoul, Republic of Korea

^4^Department of Pharmacology, College of Medicine, Kangwon National University, Chuncheon, Republic of Korea

^5^Biomedical Research Institute, Kangwon National University Hospital, Chuncheon, Republic of Korea

^#^These authors contributed equally to this article.

***Corresponding author:**

Sang-In Park, M.D., Ph.D.

Department of Pharmacology, College of Medicine, Kangwon National University, 1 Gangwondaehak-gil, Chuncheon-si, Gangwon-do 24341, Republic of Korea

Phone: +82-33-250-8851

Fax: +82-33-259-5637

E-mail: [sipark@kangwon.ac.kr](mailto:sipark@kangwon.ac.kr)

**Supplementary Table S1. Analytic sample characteristics**

|  | **Total (*n* = 486,624)** | **Missing data** |
| --- | --- | --- |
| **Age** | 56.54 (8.09) | 0 (0) |
| **Female** | 222,720 (45.8) | 0 (0) |
| **Urate (mg/dL)** | 5.20 (1.35) | 23,414 (4.81) |
| **Low-density lipoprotein cholesterol (mg/dL)** | 137.53 (33.64) | 23,722 (4.87) |
| **Triglyceride (mg/dL)** | 154.78 (91.00) | 23,226 (12.77) |
| **High-density lipoprotein cholesterol (mg/dL)** | 55.99 (14.78) | 62,132 (12.96) |
| **Total cholesterol / high-density lipoprotein cholesterol ratio** | 4.24 (1.23) | 63,047 (2.22) |
| **Gout** | 23,094 (4.7) | 0 (0) |
| **Overall ischemic diseases** | 88,843 (18.7) | 10,804 (2.22) |
| **Ischemic heart disease** | 76,219 (15.8) | 3,128 (0.64) |
| **Acute myocardial infarction** | 23,065 (5.1) | 37,602 (7.73) |
| **Cerebral infarction** | 10,346 (2.2) | 20,276 (4.17) |
| **Transient cerebral ischemic attack** | 11,496 (2.5) | 20,990 (4.31) |

Means and standard deviations are provided for continuous variables, and counts and proportions are provided for categorical variables.

**Supplementary Table** **S2. Effects of urate-lowering by *XDH-*inhibition on urate and lipid levels**

|  |  |  | **Genetic score and biomarker** | | **Genetic score and scaled urate level** | | |
| --- | --- | --- | --- | --- | --- | --- | --- |
| **Biomarker** | **Observed mean (range)** | **Residual mean (range)** | **Beta (SE)** | ***P*-value** | **Beta (SE)** | **Heterogeneity *P*-value (residual)** | **Heterogeneity *P*-value (doubly-ranked)** |
| **Urate (mg/dL)** |  |  | −1.01 (0.01) | < 0.001 | - | - | - |
| **Q1** | 3.89 (≤ 4.99) | 2.47 (< 3.03) | - | - | 8.90 (0.89) | 0.230 | 0.070 |
| **Q2** | 4.70 (3.86–5.71) | 3.39 (3.03–3.72) | - | - | 9.88 (0.38) |  |  |
| **Q3** | 5.44 (4.55–6.44) | 4.09 (3.72–4.51) | - | - | 9.77 (0.43) |  |  |
| **Q4** | 6.76 (≥ 5.45) | 5.34 (> 4.51) | - | - | 12.43 (1.46) |  |  |
| **LDL-C (mg/dL)** |  |  | −0.14 (3.21) | 0.964 | - | - | - |
| **Q1** | 96.63 (≤ 112.30) | 88.18 (< 105.57) | - | - | 11.32 (2.27) | 0.247 | 0.554 |
| **Q2** | 125.29 (115.40–135.20) | 116.91 (105.57–127.65) | - | - | 7.45 (2.15) |  |  |
| **Q3** | 146.98 (136.90–157.40) | 138.70 (127.65–150.77) | - | - | 7.92 (2.15) |  |  |
| **Q4** | 181.21 (≥ 161.30) | 172.80 (> 150.77) | - | - | 12.63 (2.14) |  |  |
| **TG (mg/dL)** |  |  | 14.26 (8.50) | 0.093 | - | - | - |
| **Q1** | 78.97 (≤ 108.90) | 2.14 (< 25.77) | - | - | 8.37 (2.01) | 0.438 | 0.135 |
| **Q2** | 110.02 (81.93–143.70) | 43.58 (25.77–62.43) | - | - | 7.80 (1.98) |  |  |
| **Q3** | 154.99 (121.43–192.60) | 87.14 (62.43–117.87) | - | - | 9.65 (2.15) |  |  |
| **Q4** | 275.15 (≥ 188.00) | 202.27 (> 117.88) | - | - | 12.48 (2.30) |  |  |
| **HDL-C (mg/dL)** |  |  | 1.70 (1.34) | 0.206 | - | - | - |
| **Q1** | 41.54 (≤ 50.62) | 43.66 (< 49.62) | - | - | 9.32 (2.44) | 0.964 | 0.635 |
| **Q2** | 49.80 (41.57–58.55) | 53.53 (49.62–57.37) | - | - | 11.03 (2.27) |  |  |
| **Q3** | 58.14 (49.23–67.25) | 61.65 (57.37–66.55) | - | - | 10.47 (2.17) |  |  |
| **Q4** | 74.49 (≥ 59.86) | 76.84 (> 66.55) | - | - | 10.57 (2.11) |  |  |
| **TC/HDL-C** |  |  | < 0.01 (0.12) | 0.991 | - | - | - |
| **Q1** | 2.99 (≤ 3.63) | 2.48 (< 2.94) | - | - | 10.45 (2.15) | 0.805 | 0.429 |
| **Q2** | 3.68 (3.16–4.30) | 3.27 (2.94–3.60) | - | - | 8.44 (2.13) |  |  |
| **Q3** | 4.42 (3.83–5.07) | 3.98 (3.60–4.43) | - | - | 10.87 (2.26) |  |  |
| **Q4** | 5.86 (≥ 4.77) | 5.37 (> 4.43) | - | - | 11.26 (2.37) |  |  |

*The ranges of observed values may overlap across quartiles because the quartiles were stratified based on residuals, not the observed values.

SE, standard error; TG, triglyceride; LDL-C, low-density lipoprotein cholesterol; HDL-C, high-density lipoprotein cholesterol; TC/HDL-C, total cholesterol/high-density lipoprotein cholesterol ratio.

**Supplementary Table S3. Information on candidate genetic instrumental variables and their proxies based on linkage disequilibrium (*r2* > 0.8; ±500 kb)**

| **Main SNP** | **rs45461698** | **rs370989010** | **rs520041** | **rs139515054** | **rs189548946** | **rs17010480** |
| --- | --- | --- | --- | --- | --- | --- |
| **Proxy SNP** | rs28493055 | n/a | rs539213, rs9751979, rs11124266, rs112980326, rs58496183, rs386605376, rs809366 |  | rs192431122, rs192745559 | rs62139536, rs17010474, rs10205350, rs52801909 |
| **PhenoScanner** | Cause of death: myelodysplastic syndrome, unspecified  Cause of death: cholangitis | n/a | *NLRC4* gene expression  IL-18 protein expression  *SLC30A6* gene expression  *YIPF4* gene expression | n/a | n/a | *CAPN13* gene expression |
| **GWAS Catalog** | n/a | n/a | n/a | Urate | n/a | n/a |
| **GTExPortal** | n/a | NLRC4 gene expression (cells - cultured fibroblasts) | n/a | n/a | n/a | n/a |
| **Decision** | Included | Excluded | Excluded | Included | Included | Excluded |

Only associations at *P* < 5 × 10^−8^ were considered.

SNP, single nucleotide polymorphism

**Supplementary Table S4. Associations between genetic instruments and negative control outcomes**

|  | **Negative control outcome** | | | |
| --- | --- | --- | --- | --- |
|  | **Age** |  | **Biological sex** |  |
| **Genetic instrument** | **Beta (SE)** | ***P*-value** | **Beta (SE)** | ***P*-value** |
| rs45461698 | 0.04 (0.08) | 0.609 | -0.02 (0.02) | 0.415 |
| rs139515054 | 0.03 (0.13) | 0.788 | 0.01 (0.03) | 0.846 |
| rs189548946 | 0.05 (0.15) | 0.739 | 0.04 (0.04) | 0.222 |
| Genetic score | 0.23 (0.75) | 0.759 | -0.20 (0.19) | 0.279 |

SE, standard error.

**Supplementary Table S5. Linear MR estimates and sensitivity analyses for horizontal pleiotropy**

| **Disease** | **Wald ratio** | | **Wald ratio adjusting for BMI** | | **MR-cML** | |
| --- | --- | --- | --- | --- | --- | --- |
|  | **OR (95% CI)** | ***P*-value** | **OR (95% CI)** | ***P*-value** | **OR (95% CI)** | ***P*-value** |
| Gout | 0.85 (0.78–0.93) | <0.001 | 0.85 (0.77–0.93) | <0.001 | 0.83 (0.73–0.95) | 0.005 |
| Cerebral infarction | 0.86 (0.75–0.98) | 0.023 | 0.86 (0.75–0.99) | 0.031 | 0.85 (0.73, 0.98) | 0.030 |
| Acute myocardial infarction | 0.98 (0.89–1.07) | 0.607 | 0.98 (0.89–1.07) | 0.629 | 0.97 (0.88–1.08) | 0.613 |
| Ischemic heart disease | 0.99 (0.94–1.04) | 0.667 | 0.99 (0.93–1.04) | 0.650 | 0.99 (0.93–1.05) | 0.694 |
| Transient cerebral ischemic attack | 0.98 (0.87–1.11) | 0.768 | 0.98 (0.86­–1.12) | 0.781 | 0.98 (0.85–1.12) | 0.742 |
| Overall ischemic disease | 0.97 (0.93–1.02) | 0.297 | 0.97 (0.92–1.03) | 0.306 | 0.97 (0.92–1.03) | 0.326 |

MR, Mendelian randomization; BMI, body mass index; cML, constrained maximum likelihood; OR, odds ratio; CI, confidence interval.

**Supplementary Table S6. Nonlinear MR for gout and overall ischemic disease**

|  | **Gout** | | | **Overall ischemic disease** | | |
| --- | --- | --- | --- | --- | --- | --- |
|  | **OR** | **95% CI** | | **OR** | **95% CI** | |
|  |  | **Lower** | **Upper** |  | **Lower** | **Upper** |
| **Urate** |  |  |  |  |  |  |
| **Q1** | 0.85 | 0.68 | 1.08 | 0.94 | 0.85 | 1.05 |
| **Q2** | 0.86 | 0.66 | 1.12 | 0.98 | 0.88 | 1.09 |
| **Q3** | 0.86 | 0.68 | 1.08 | 1 | 0.9 | 1.11 |
| **Q4** | 0.87 | 0.76 | 0.99 | 0.97 | 0.88 | 1.07 |
| ***P* (heterogeneity)** | 0.999 |  |  | 0.884 |  |  |
| ***P* (trend)** | 0.902 |  |  | 0.745 |  |  |
| **TG** |  |  |  |  |  |  |
| **Q1** | 0.89 | 0.71 | 1.11 | 1.02 | 0.91 | 1.14 |
| **Q2** | 0.77 | 0.61 | 0.96 | 0.96 | 0.86 | 1.08 |
| **Q3** | 0.84 | 0.69 | 1.02 | 0.98 | 0.88 | 1.09 |
| **Q4** | 0.9 | 0.76 | 1.06 | 0.93 | 0.84 | 1.04 |
| ***P* (heterogeneity)** | 0.719 |  |  | 0.734 |  |  |
| ***P* (trend)** | 0.535 |  |  | 0.326 |  |  |
| **LDL-C** |  |  |  |  |  |  |
| **Q1** | 0.82 | 0.7 | 0.97 | 0.95 | 0.86 | 1.04 |
| **Q2** | 0.88 | 0.73 | 1.07 | 0.98 | 0.88 | 1.09 |
| **Q3** | 0.91 | 0.75 | 1.12 | 0.94 | 0.84 | 1.06 |
| **Q4** | 0.83 | 0.68 | 1.01 | 1.02 | 0.92 | 1.13 |
| ***P* (heterogeneity)** | 0.845 |  |  | 0.736 |  |  |
| ***P* (trend)** | 0.899 |  |  | 0.397 |  |  |
| **HDL-C** |  |  |  |  |  |  |
| **Q1** | 0.83 | 0.7 | 0.98 | 0.9 | 0.82 | 0.99 |
| **Q2** | 0.86 | 0.72 | 1.02 | 0.98 | 0.88 | 1.08 |
| **Q3** | 0.95 | 0.78 | 1.15 | 1.12 | 1.01 | 1.25 |
| **Q4** | 0.86 | 0.69 | 1.06 | 0.95 | 0.84 | 1.06 |
| ***P* (heterogeneity)** | 0.776 |  |  | 0.023 |  |  |
| ***P* (trend)** | 0.702 |  |  | 0.333 |  |  |
| **TC/HDL-C** |  |  |  |  |  |  |
| **Q1** | 0.79 | 0.64 | 0.96 | 0.97 | 0.87 | 1.08 |
| **Q2** | 0.98 | 0.8 | 1.2 | 1.07 | 0.96 | 1.19 |
| **Q3** | 0.83 | 0.68 | 1 | 0.93 | 0.84 | 1.04 |
| **Q4** | 0.89 | 0.75 | 1.05 | 0.96 | 0.87 | 1.06 |
| ***P* (heterogeneity)** | 0.442 |  |  | 0.335 |  |  |
| ***P* (trend)** | 0.686 |  |  | 0.497 |  |  |

MR, Mendelian randomization; OR, odds ratio; CI, confidence interval; TG, triglyceride; LDL-C, low-density lipoprotein cholesterol; HDL-C, high-density lipoprotein cholesterol; TC/HDL-C, total cholesterol/high-density lipoprotein cholesterol ratio.

**Supplementary Table S7. Nonlinear MR for acute myocardial infarction and cerebral infarction**

|  | **Acute myocardial infarction** | | | **Cerebral infarction** | | |
| --- | --- | --- | --- | --- | --- | --- |
|  | **OR** | **95% CI** | | **OR** | **95% CI** | |
|  |  | **Lower** | **Upper** |  | **Lower** | **Upper** |
| **Urate** |  |  |  |  |  |  |
| **Q1** | 0.91 | 0.75 | 1.11 | 0.91 | 0.69 | 1.19 |
| **Q2** | 0.97 | 0.8 | 1.19 | 0.95 | 0.7 | 1.27 |
| **Q3** | 1.02 | 0.84 | 1.23 | 0.86 | 0.65 | 1.13 |
| **Q4** | 0.93 | 0.79 | 1.11 | 0.76 | 0.59 | 0.98 |
| ***P* (heterogeneity)** | 0.354 |  |  | 0.697 |  |  |
| ***P* (trend)** | 0.854 |  |  | 0.278 |  |  |
| **TG** |  |  |  |  |  |  |
| **Q1** | 1.04 | 0.85 | 1.26 | 0.75 | 0.57 | 1 |
| **Q2** | 0.96 | 0.77 | 1.19 | 0.95 | 0.71 | 1.28 |
| **Q3** | 1.1 | 0.91 | 1.35 | 0.98 | 0.74 | 1.3 |
| **Q4** | 0.79 | 0.66 | 0.94 | 0.78 | 0.59 | 1.03 |
| ***P* (heterogeneity)** | 0.069 |  |  | 0.458 |  |  |
| ***P* (trend)** | 0.031 |  |  | 0.763 |  |  |
| **LDL-C** |  |  |  |  |  |  |
| **Q1** | 0.93 | 0.81 | 1.08 | 0.76 | 0.61 | 0.96 |
| **Q2** | 0.83 | 0.67 | 1.03 | 0.99 | 0.75 | 1.32 |
| **Q3** | 1.05 | 0.83 | 1.32 | 0.67 | 0.49 | 0.92 |
| **Q4** | 1.03 | 0.84 | 1.27 | 1.15 | 0.87 | 1.54 |
| ***P* (heterogeneity)** | 0.41 |  |  | 0.037 |  |  |
| ***P* (trend)** | 0.331 |  |  | 0.088 |  |  |
| **HDL-C** |  |  |  |  |  |  |
| **Q1** | 0.87 | 0.75 | 1.02 | 0.81 | 0.64 | 1.03 |
| **Q2** | 0.95 | 0.8 | 1.13 | 0.96 | 0.74 | 1.25 |
| **Q3** | 1.13 | 0.93 | 1.38 | 0.88 | 0.67 | 1.17 |
| **Q4** | 0.99 | 0.78 | 1.26 | 0.8 | 0.59 | 1.09 |
| ***P* (heterogeneity)** | 0.246 |  |  | 0.765 |  |  |
| ***P* (trend)** | 0.186 |  |  | 0.841 |  |  |
| **TC/HDL-C** |  |  |  |  |  |  |
| **Q1** | 0.89 | 0.74 | 1.07 | 0.8 | 0.61 | 1.05 |
| **Q2** | 1.16 | 0.95 | 1.41 | 0.95 | 0.71 | 1.26 |
| **Q3** | 0.94 | 0.78 | 1.14 | 0.8 | 0.61 | 1.06 |
| **Q4** | 0.9 | 0.75 | 1.07 | 0.95 | 0.73 | 1.24 |
| ***P* (heterogeneity)** | 0.188 |  |  | 0.692 |  |  |
| ***P* (trend)** | 0.569 |  |  | 0.513 |  |  |

MR, Mendelian randomization; OR, odds ratio; CI, confidence interval; TG, triglyceride; LDL-C, low-density lipoprotein cholesterol; HDL-C, high-density lipoprotein cholesterol; TC/HDL-C, total cholesterol/high-density lipoprotein cholesterol ratio.

**Supplementary Table** **S8. Nonlinear MR for ischemic heart disease and transient cerebral ischemic attack**

|  | **Ischemic heart disease** | | | **Transient cerebral ischemic attack** | | |
| --- | --- | --- | --- | --- | --- | --- |
|  | **OR** | **95% CI** | | **OR** | **95% CI** | |
|  |  | **Lower** | **Upper** |  | **Lower** | **Upper** |
| **Urate** |  |  |  |  |  |  |
| **Q1** | 0.94 | 0.84 | 1.05 | 1.03 | 0.8 | 1.32 |
| **Q2** | 0.97 | 0.86 | 1.09 | 1.02 | 0.78 | 1.33 |
| **Q3** | 1.04 | 0.93 | 1.16 | 0.82 | 0.63 | 1.07 |
| **Q4** | 1 | 0.91 | 1.11 | 1.04 | 0.82 | 1.32 |
| ***P* (heterogeneity)** | 0.658 |  |  | 0.532 |  |  |
| ***P* (trend)** | 0.37 |  |  | 0.937 |  |  |
| **TG** |  |  |  |  |  |  |
| **Q1** | 1.07 | 0.95 | 1.21 | 0.9 | 0.69 | 1.18 |
| **Q2** | 0.98 | 0.87 | 1.11 | 0.79 | 0.6 | 1.04 |
| **Q3** | 0.96 | 0.86 | 1.08 | 1.06 | 0.81 | 1.38 |
| **Q4** | 0.95 | 0.85 | 1.05 | 1.16 | 0.9 | 1.5 |
| ***P* (heterogeneity)** | 0.444 |  |  | 0.184 |  |  |
| ***P* (trend)** | 0.2 |  |  | 0.067 |  |  |
| **LDL-C** |  |  |  |  |  |  |
| **Q1** | 0.96 | 0.87 | 1.06 | 0.95 | 0.78 | 1.16 |
| **Q2** | 0.97 | 0.86 | 1.09 | 1.13 | 0.86 | 1.48 |
| **Q3** | 1.03 | 0.92 | 1.17 | 0.75 | 0.56 | 1.02 |
| **Q4** | 1 | 0.89 | 1.12 | 1.14 | 0.86 | 1.51 |
| ***P* (heterogeneity)** | 0.82 |  |  | 0.152 |  |  |
| ***P* (trend)** | 0.525 |  |  | 0.62 |  |  |
| **HDL-C** |  |  |  |  |  |  |
| **Q1** | 0.89 | 0.81 | 0.98 | 1.02 | 0.82 | 1.28 |
| **Q2** | 1.01 | 0.91 | 1.12 | 1.03 | 0.81 | 1.32 |
| **Q3** | 1.14 | 1.02 | 1.27 | 0.99 | 0.76 | 1.28 |
| **Q4** | 0.99 | 0.87 | 1.12 | 0.84 | 0.63 | 1.11 |
| ***P* (heterogeneity)** | 0.016 |  |  | 0.676 |  |  |
| ***P* (trend)** | 0.11 |  |  | 0.255 |  |  |
| **TC/HDL-C** |  |  |  |  |  |  |
| **Q1** | 0.98 | 0.88 | 1.1 | 0.83 | 0.65 | 1.06 |
| **Q2** | 1.09 | 0.97 | 1.22 | 1.1 | 0.85 | 1.42 |
| **Q3** | 0.97 | 0.86 | 1.08 | 0.99 | 0.76 | 1.28 |
| **Q4** | 0.95 | 0.85 | 1.05 | 1.06 | 0.82 | 1.37 |
| ***P* (heterogeneity)** | 0.297 |  |  | 0.4 |  |  |
| ***P* (trend)** | 0.181 |  |  | 0.292 |  |  |

MR, Mendelian randomization; OR, odds ratio; CI, confidence interval; TG, triglyceride; LDL-C, low-density lipoprotein cholesterol; HDL-C, high-density lipoprotein cholesterol; TC/HDL-C, total cholesterol/high-density lipoprotein cholesterol ratio

**Supplementary Table S9. Case and control statuses based on ICD-10 codes**

|  | **Case**  **(ICD-10 code)** | **Control exclusion (Phecode version 1.2)**  **(ICD-10 code)** |
| --- | --- | --- |
| **Gout** | M10 | M10 |
| **Acute myocardial infarction** | I21, I22, I23 | I20, I21, I22, I23, I24, I25, I34.1, I46.1, I51.0, I51.1, I51.2, I51.3, I51.5, I51.8, I51.9, I52, Z95.1, Z95.5 |
| **Ischemic heart disease** | I20–I25 |  |
| **Cerebral infarction** | I63 | G45.0, G45.1, G45.2, G45.4, G45.8, G45.9, G46.0, G46.1, G46.2, G46.3, G46.4, G46.5, G46.6, G46.7, G46.8, I60, I61, I62.0, I62.1, I62.9, I63, I64, I65, I66, I67, I68, I69, S06.4 |
| **Transient cerebral ischemic attack** | G45 |  |
| **Overall ischemic disease** | I20–I25, I63, G45 | G46.0, G46.1, G46.2, G46.3, G46.4, G46.5, G46.6, G46.7, G46.8, I34.1, I46.1, I51.0, I51.1, I51.2, I51.3, I51.5, I51.8, I51.9, I52, I60, I61, I62.0, I62.1, I62.9, I64, I65, I66, I67, I68, I69, S06.4, Z95.1, Z95.5 |

ICD-10, International Classification of Diseases 10^th^ Revision
